# Cortical Hand Knob Stroke: A Case Report of Isolated Hand Weakness and Recovery

**DOI:** 10.7759/cureus.73840

**Published:** 2024-11-17

**Authors:** Sharafath Hussain Zahir Hussain, Maria Elizabeth Vincent, Emil Tom John, Fatima Ihsan

**Affiliations:** 1 Internal Medicine, Milton Keynes University Hospital, Milton Keynes, GBR

**Keywords:** cerebrovascular accident (stroke), cortical hand, cortical hand knob stroke, general internal medicine, peripheral neuropathy, stroke, sudden weakness

## Abstract

Cortical hand knob stroke is a rare form of stroke that affects the motor cortex responsible for controlling fine hand movements. This condition, most commonly caused by ischemia in the "hand knob" region of the precentral gyrus, can present with isolated hand weakness, often mimicking peripheral neuropathies and leading to diagnostic delays. We report a case of a 65-year-old right-handed woman who experienced a sudden onset of left-hand weakness, along with resolving slurred speech and facial droop, while she was working at her office. Due to her persistent left-hand weakness, magnetic resonance imaging of the brain was performed. This revealed a focal ischemic lesion in the right precentral gyrus, specifically involving the cortical hand knob region. The patient was treated with antiplatelet therapy, high-dose statins, and lifestyle modifications, including smoking cessation. Over five months, she showed significant recovery of hand strength. This case highlights the diagnostic challenge posed by cortical hand knob stroke, which is often misinterpreted as peripheral neuropathy, leading to delays in appropriate treatment. Recognizing this rare stroke subtype is essential for timely management and improved patient outcomes.

## Introduction

Cortical strokes involving the "hand knob" region of the precentral gyrus represent a rare but clinically significant subtype of stroke, characterized by isolated motor deficits that primarily affect fine motor control of the hand. The "hand knob" is anatomically identified as a knob-like projection of the superior precentral gyrus, corresponding to the middle genu of the central sulcus, and appears as an omega-shaped structure on axial brain images [1]. This distinct morphology and location make it a reliable landmark for identifying the motor hand area. Functionally, the hand knob region serves as the origin of critical motor pathways, including the corticospinal, corticobulbar, and cortico-rubro-spinal tracts [2]. Ischemic damage to this area often presents as isolated unilateral hand weakness or pure motor monoparesis [3]. Differential diagnoses upon presentation for cortical hand knob stroke include peripheral neuropathies such as carpal tunnel syndrome or radial nerve palsy. Additionally, other central nervous system causes, including lesions in the parietal lobe, ventroposterior thalamus, and posterior limb of the internal capsule, should be considered when evaluating isolated hand weakness. These overlapping clinical features can pose diagnostic challenges, delaying appropriate treatment [2].

Epidemiological studies indicate that hand knob strokes are exceptionally rare, accounting for less than 1% of all ischemic strokes [2]. Studies, including that by de Medeiros et al. [1] and Orosz et al. [2], have shown that these strokes more frequently affect the right-hand knob (56%) than the left (40%). Embolism is reported as the most common etiology, though other mechanisms have also been implicated [2]. Accurate diagnosis often relies on imaging techniques to identify the central sulcus and distinguish hand knob lesions from other causes of isolated hand weakness as described above [3]. The majority of the cases of hand knob stroke have a good functional outcome secondary to neuronal plasticity, and the risk of recurrence is low. The rarity of the presentation poses challenges in terms of data acquisition regarding the clinical course [4]. Management of cortical hand knob stroke follows standard acute ischemic stroke protocols, including timely thrombolysis or thrombectomy when indicated, along with antiplatelet therapy and secondary prevention measures such as high-dose statins, blood pressure control, and lifestyle modifications. The principle of "time is brain" highlights the importance of early intervention and improved patient outcomes [5]. The typical patient loses 1.9 million neurons each minute in which stroke is untreated [5].

In this case report, we discuss a 65-year-old right-handed woman who experienced sudden left-hand weakness, along with transient slurred speech and facial droop, while at her office. MRI findings revealed a focal ischemic lesion in the right precentral gyrus, specifically involving the hand knob area. This case highlights the diagnostic challenges associated with cortical hand knob strokes, which may be misinterpreted as peripheral neuropathy.

## Case presentation

A 65-year-old right-handed woman with a history of hypertension, hypothyroidism, and a 20-pack-year smoking history initially presented to the emergency department 10 hours after symptom onset with resolving left-sided facial droop, slurred speech, and persistent left-hand grip weakness. She exhibited an initial left-hand strength of 3/5 during the neurological examination, and no other motor deficits were observed. Upon review by the stroke team, her facial weakness and slurred speech had resolved, and her hand grip strength had improved to 4/5. The clinical impression was a transient ischemic attack (TIA) due to her resolving clinical picture. She was subsequently loaded with aspirin 300 mg and referred to the TIA clinic. At the clinic, she reported residual left-hand grip weakness, which was particularly distressing due to the impact it had on her work as her job required frequent typing. Vital signs on presentation included a blood pressure of 165/95 mmHg, with heart rate, oxygen saturation, and respiratory rate within normal ranges. She had isolated minor motor weakness, as indicated by her National Institutes of Health Stroke Scale (NIHSS) score of 1.

Initial investigations included a carotid Doppler ultrasound, which revealed moderate stenosis (50-69%) of the left internal carotid artery and mild stenosis (1-19%) of the right internal carotid artery. An electrocardiogram (ECG) demonstrated normal sinus rhythm with no evidence of atrial fibrillation, and a 2D echocardiogram showed no structural abnormalities. Blood tests indicated elevated cholesterol levels, with total cholesterol of 265 mg/dL (6.86 mmol/L) and low-density lipoprotein cholesterol of 190 mg/dL (4.92 mmol/L); however, thyroid function tests and other metabolic parameters were normal.

**Figure 1:**
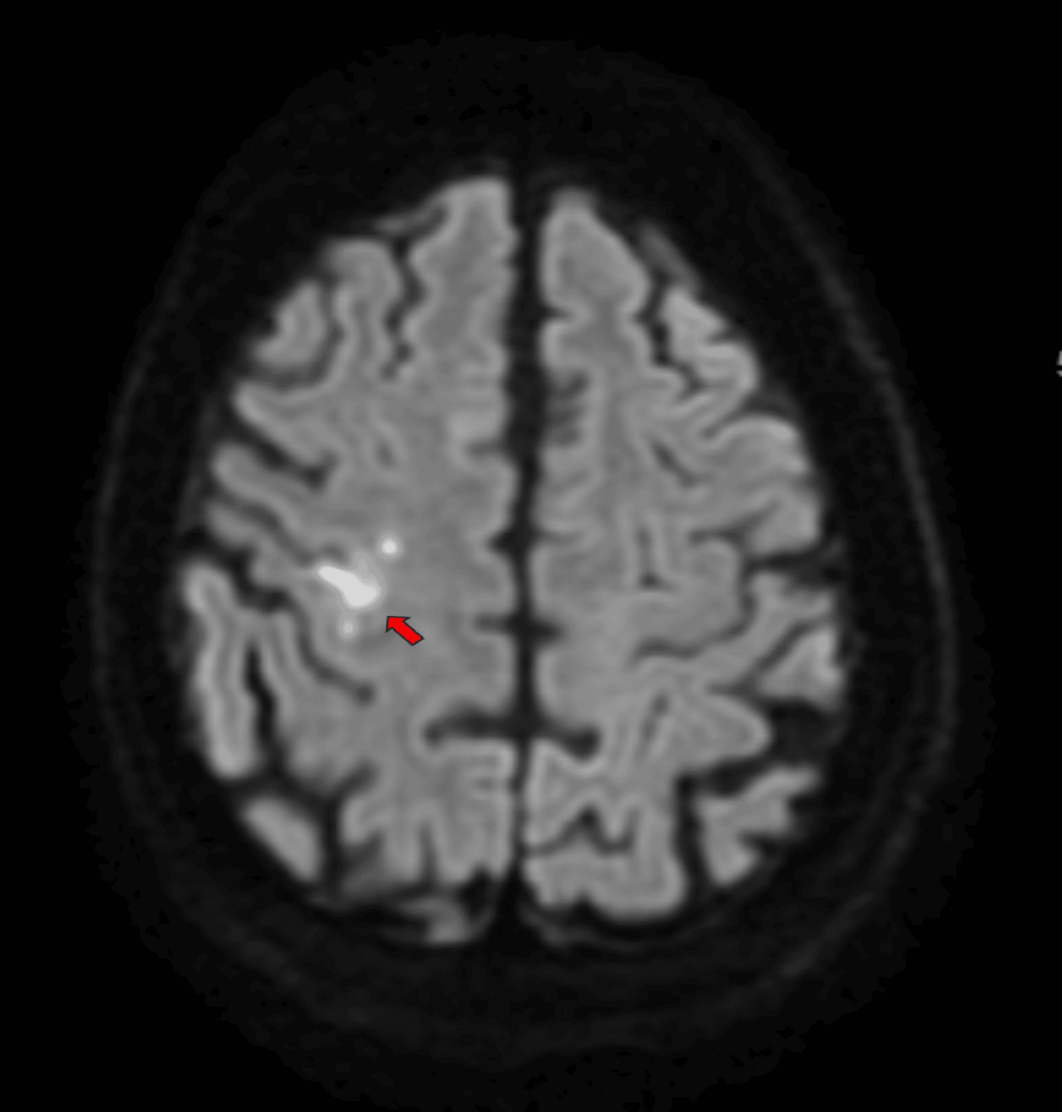
MRI of the brain showing a small acute infarct in the right precentral gyrus, specifically involving the hand knob area. There are a number of small regions of restricted diffusion on the right-hand knob area on a background of small vessel disease.

Given the persistent left-hand weakness and her vascular risk factors, neuroimaging was requested. Magnetic resonance imaging (MRI) of the brain revealed a small acute infarct in the right precentral gyrus, specifically involving the hand knob area, as indicated in Figure 1. This lesion appeared hyperintense on T2-weighted and fluid-attenuated inversion recovery (FLAIR) sequences, with no evidence of hemorrhage or mass effect. Diffusion-weighted imaging (DWI) confirmed acute ischemia in the same region, correlating with her clinical symptoms.

The final impression was a cortical hand knob stroke. She was initiated on a loading dose of aspirin (300 mg) for two weeks and transitioned to long-term antiplatelet therapy with clopidogrel (75 mg daily). Due to her elevated cholesterol levels and as secondary prevention, high-dose atorvastatin (80 mg) was initiated. Her antihypertensive treatment was optimized with amlodipine (10 mg) to achieve better blood pressure control, and she was strongly advised to quit smoking as part of her lifestyle modification plan to reduce the risk of future vascular events.

The patient was monitored closely over the following months, showing a gradual improvement in the strength of her left hand. She attended regular physiotherapy sessions, and by the fifth month post stroke, she reported nearly complete recovery of her hand function.

## Discussion

Cortical hand knob stroke is a rare but clinically significant type of stroke that occurs at the hand knob region within the precentral gyrus, a motor control area responsible for hand movements [1]. Major motor pathways, including the corticospinal, corticobulbar, and cortico-rubro-spinal tracts, originate here. Anatomically, the hand knob is a knob-like projection of the precentral gyrus that corresponds to the middle genu of the central sulcus, and it serves as a reliable landmark for identifying the motor hand area. Consistently described as an omega-shaped structure in axial brain images, this anatomical description is widely recognized [3]. Although uncommon, accounting for less than 1% of ischemic strokes, its clinical presentation often mimics peripheral nerve injuries like carpal tunnel syndrome or ulnar neuropathy due to the isolated nature of hand weakness [2].

In 1997, Yousry et al. reported the first case of a patient with a circumscribed infarct in the "hand knob area," presenting with isolated contralateral arm weakness without any sensory changes [4]. These subsets of patients may not exhibit typical pyramidal signs or other cortical involvement and thus pose a challenge in differentiating from peripheral neuropathy, leading to misdiagnosis and delayed treatment [4]. These small cortical strokes often lead to weakness limited to specific finger groups, a phenomenon termed “pseudo-peripheral palsy.” Imaging studies have demonstrated that such presentations are linked to lesions in the hand knob area, predominantly affecting muscles innervated by the ulnar or radial nerves [6]. To date, no reports are available about strokes leading to paresis of the muscles exclusively controlled by the median nerve [7]. It is essential to consider cerebral infarction as a differential diagnosis in patients with vascular risk factors presenting with sensory-motor deficits that mimic isolated peripheral nerve injuries. Reflecting on our case, the presentation of a resolving TIA accompanied by isolated hand weakness served as a key clue to the diagnostic challenge.

The etiology of cortical hand knob stroke is often linked to embolic events. Studies suggest that both artery-to-artery emboli and cardioembolic mechanisms contribute to this condition [2,8], with atherosclerosis frequently identified as a common underlying cause. Recent research has highlighted large artery atherosclerosis (LAA) and small vessel occlusion (SVO) as common mechanisms behind hand knob strokes. Zhang et al. (2022) found LAA and SVO to be the leading causes in their case series, with hypertension being the most prevalent risk factor, followed by hyperlipidemia and hyperhomocysteinemia [8]. Similarly, Orosz et al. (2018) observed that significant carotid artery stenosis was common among their patients, further underscoring the role of embolic sources in the pathogenesis of this stroke subtype [2]. In our case, the patient’s significant risk factors, including hypertension, hyperlipidemia, and smoking, align with risk profiles observed in other cases of hand knob stroke, with small vessel disease as a likely underlying etiology [2,8]. While embolic infarction remains the primary cause, literature also documents instances where isolated upper extremity weakness resulted from cerebral metastasis affecting the hand knob area [9]. Monoparesis caused by cerebral insult of vascular causes is rare and is usually caused by mass lesions or other focal pathology [7].

Brigo et al. (2018) proposed a clinical approach using synkinetic wrist extension to differentiate cortical hand strokes from peripheral nerve palsy. They noted that patients with central wrist drop tend to preserve synkinetic wrist extension of the forearm extensors when making a fist, causing a slight elevation of the hand. In contrast, clenching the fist with a peripheral nerve lesion results in a worsening of the wrist drop, providing a distinguishing feature between the two conditions [10]. Additionally, the onset of symptoms, along with variations in sensation and reflexes, can further assist in differentiation. Cortical strokes typically preserve sensory function and are often associated with hyperreflexia, whereas peripheral nerve palsies tend to present with sensory deficits and diminished or absent reflexes in the affected region. The sudden onset of symptoms, combined with preserved sensation, played a crucial role in reaching the diagnosis in our patient.

Most cases of hand knob stroke have a positive prognosis, with good functional recovery due to neuronal plasticity and a low risk of recurrence. This outcome is particularly likely with early diagnosis and proper management. Studies, including those by Orosz et al. (2018) and Zhang et al. (2022), have shown that most patients experience good recovery of hand function with minimal long-term deficits [2,8]. This favorable outcome may be partly due to the unique blood supply of the precentral gyrus, which is supplied by both the anterior cerebral artery (ACA) and the middle cerebral artery (MCA). The superior division of the MCA supplies the lateral surface of the precentral gyrus, while the ACA supplies the medial portion. This dual supply creates an anastomotic network that provides collateral blood flow, which can help preserve tissue function even when part of the MCA or one of its branches is compromised [11]. In our case, the patient showed significant improvement in hand strength over several months, aligning with the literature that suggests the hand knob region's abundant collateral blood supply may contribute to better outcomes [2,8].

Some case reports describe the effectiveness of early physiotherapy in individuals with stroke of the hand knob area presenting with isolated wrist drops. These reports suggest that targeted physiotherapy helps improve motor recovery by preventing compensatory movements, ultimately leading to better overall outcomes in patients with hand knob area strokes [12].

## Conclusions

Cortical hand knob stroke, although rare, should be a key consideration in patients presenting with isolated hand weakness, especially in those with vascular risk factors. This case report of a 65-year-old woman illustrates the diagnostic challenges associated with this condition, which can often be misinterpreted as peripheral neuropathy. Timely and accurate identification of cortical hand knob strokes is critical to initiating appropriate management, including antiplatelet therapy and lifestyle modifications.

Our patient demonstrated significant improvement in hand strength over five months, consistent with literature suggesting favorable outcomes associated with this stroke subtype. Early intervention and targeted rehabilitation can enhance recovery and functional outcomes, highlighting the importance of recognizing and understanding the unique characteristics of cortical hand knob strokes. Without proper diagnosis, there is a risk of misclassifying central motor neuron lesions as peripheral conditions, potentially delaying effective treatment and impacting patient recovery.

## References

[1] de Medeiros FC, Viana DC, Cunha MN, Hatasa CC, Araújo RV (2017). Pure motor monoparesis due to infarction of the "hand knob" area: radiological and morphological features. Neurol Sci.

[2] Orosz P, Szőcs I, Rudas G, Folyovich A, Bereczki D, Vastagh I (2018). Cortical hand knob stroke: report of 25 cases. J Stroke Cerebrovasc Dis.

[3] Kaneko OF, Fischbein NJ, Rosenberg J, Wintermark M, Zeineh MM (2017). The “white gray sign” identifies the central sulcus on 3T high-resolution T1-weighted images. AJNR Am J Neuroradiol.

[4] Rissardo JP, Byroju VV, Mukkamalla S, Caprara AL (2024). A narrative review of stroke of cortical hand Knob area. Medicina (Kaunas).

[5] Saver JL (2006). Time is brain--quantified. Stroke.

[6] Wang Y, Dong Q, Li SJ, Hu WL (2018). New clinical characteristics and risk factors of hand knob infarction. Neurol Sci.

[7] Tahir H, Daruwalla V, Meisel J, Kodsi SE (2016). Pseudoradial nerve palsy caused by acute ischemic stroke. J Investig Med High Impact Case Rep.

[8] Zhang Z, Sun X, Liu X, Wang L, Zhu R (2022). Clinical features, etiology, and prognosis of hand knob stroke: a case series. BMC Neurol.

[9] Folyovich A, Varga V, Várallyay G (2018). A case report of isolated distal upper extremity weakness due to cerebral metastasis involving the hand knob area. BMC Cancer.

[10] Brigo F, Ragnedda G, Canu P, Nardone R (2018). Synkinetic wrist extension in distinguishing cortical hand from radial nerve palsy. Pract Neurol.

[11] Kesserwani H (2020). Ischemic infarct of the hand knob gyrus: natural history, morphology, and localizing value of the omega sulcus - a case report with a side note on the dynamic forces underlying sulci formation. Cureus.

[12] Jain M, Harjpal P, Kovela RK, Vardhan V (2022). Positive outcomes of early task-specific training and action observation mirror therapy following infarction of hand knob area: a case report. Cureus.

